# HNRNPA2B1: a novel target in pulmonary arterial hypertension

**DOI:** 10.3389/fcvm.2025.1497938

**Published:** 2025-07-09

**Authors:** Yingying Wei, Daiqin Wu, Na Deng, Fujia Xu, Sihan Luo, Xinxin Fan, Haijun Guo, Jingjing Chen, Wei Li, Xiaoyun Si

**Affiliations:** Department of Cardiovascular Medicine, Affiliated Hospital of Guizhou Medical University, Guiyang, China

**Keywords:** pulmonary arterial hypertension, HNRNPA2B1, vascular remodeling, smooth muscle cells, endothelial cell

## Abstract

**Purpose of Review:**

Pulmonary arterial hypertension (PAH) is a progressive clinical syndrome characterized by pulmonary vascular remodeling and elevated pulmonary artery pressure, associated with high morbidity and mortality. While targeted therapies have improved patient prognosis, restoring normal hemodynamics and reversing vascular pathology remain unmet challenges. Heterogeneous nuclear ribonucleoprotein A2/B1 (HNRNPA2B1), an RNA-binding protein integral to mRNA processing and post-transcriptional regulation, governs critical processes including cell proliferation, apoptosis, angiogenesis, and endothelial homeostasis. However, its role in PAH pathogenesis remains poorly defined. This review synthesizes current evidence on HNRNPA2B1 in PAH, evaluates its potential mechanistic contributions, and discusses therapeutic implications. Given the fact that much of the connections between PAH and HNRNPA2B1 are speculative, rigorous mechanistic studies are imperative to clarify its pathobiological relevance.

**Recent Findings:**

Emerging preclinical evidence suggests that HNRNPA2B1 silencing attenuates monocrotaline (MCT)-induced pulmonary hypertension (PH) in rat models. Mechanistically, HNRNPA2B1 modulates vascular smooth muscle cell (VSMC) proliferation via cross-talk between multiple signaling cascades and macrophage polarization dynamics, both central to pulmonary vascular remodeling. Nevertheless, clinical translatability remains uncertain, as no studies have yet conclusively validated HNRNPA2B1 as a druggable target in human PAH.

**Summary:**

Recent evidence suggests HNRNPA2B1 has emerged as a potential therapeutic target for PAH. However, further studies are essential to elucidate its role in modulating the pathogenic mechanisms underlying PAH.

## 1 Introduction

Pulmonary hypertension (PH) is a progressive disorder characterized by elevated pulmonary arterial pressure and vascular remodeling, culminating in right heart failure and premature mortality. Globally, PH imposes a significant economic burden, with an estimated prevalence affecting approximately 1% of the population (1).

PH encompasses five distinct clinical subtypes: group 1 pulmonary arterial hypertension (PAH), group 2 PH associated with left heart disease (PH-LHD), group 3 PH associated with lung diseases and/or hypoxia, group 4 PH associated with pulmonary artery obstruction, and group 5 PH with unclear and/or multifactorial mechanisms. The pathogenesis of PAH is intricately multifaceted, involving a spectrum of mechanisms, such as endothelial cell dysfunction, aberrant smooth muscle cell proliferation, plexiform lesions, inflammation, immune responses, cytokine activity, chronic thrombosis, small vessel occlusion, anti-apoptotic processes, as well as metabolic and hormonal influences (2). Currently, the most commonly studied animal models primarily replicate Group 1 and Group 3 PH. This review primarily focuses on Group 1 PH. Patients with PAH are hemodynamically defined by pre-capillary PH, except in cases of other underlying causes of pre-capillary PH (1). Although targeted therapies have significantly improved the prognosis of PAH patients, it remains a challenge to reduce pulmonary arterial pressure to normal levels and reverse pulmonary vascular remodeling. Targeted therapies for PAH primarily target three pathways: endothelin receptors on pulmonary arterial smooth muscle cells (PASMCs), which promote vasoconstriction and cell proliferation; the nitric oxide-induced activation of soluble guanylate cyclase (sGC); and the prostacyclin metabolic pathway. Therefore, calcium channel blockers are used to treat PAH patients who show a positive response to acute vasoreactivity testing (1). There are also new drugs under clinical investigation that target other pathways and have been reported to improve patient symptoms and hemodynamics (2)^.^ With these therapies, 5-year survival improved by more than 60% in 2015 (3). However, these drugs cannot reverse vascular remodeling, although they function by inducing vasodilation to alleviate clinical symptoms. Therefore, it is crucial to explore the pathophysiological mechanisms of PAH in order to provide early interventions and improve prognosis.

Heterogeneous nuclear ribonucleoprotein A2/B1 (HNRNPA2B1) is an RNA-binding protein (RBP) that regulates the mRNA expression of several genes and exhibits diverse biological effects, making it a potential therapeutic target for multiple diseases.

The role of HNRNPA2B1 has primarily been studied in the field of cancer. The mechanisms driving PAH development partially overlap with those of carcinogenesis. Cell proliferation and anti-apoptotic phenotypes are also key features of PAH. HNRNPA2B1 can promote cell proliferation and prevent apoptosis through various pathways, and it can also promote tumor cell proliferation through mechanisms such as epithelial-mesenchymal transition (EMT) and angiogenesis (4, 5). However, its role in PAH was not elucidated until 2022. Silencing of HNRNPA2B1 can mitigate monocrotaline (MCT)-induced PAH in rats (6). Moreover, HNRNPA2B1 is upregulated and localized in the nucleus of patients with idiopathic pulmonary hypertension (IPAH), where it participates in the development of PAH by regulating the cell cycle (6). However, the detailed mechanisms remain under investigation. HNRNPA2B1 has been reported to promote proliferation and prevent apoptosis by activating the STAT3 and Erk1/2 signaling pathways (7). Importantly, these two signaling pathways are also crucial for promoting proliferation and preventing apoptosis in PASMCs (8, 9). Furthermore, HNRNPA2B1 regulates the maturation of mRNA precursors by recognizing the N6-methyladenosine (m6A) post-transcriptional modification (10). It is also associated with the exosomal secretion of miRNAs in endothelial cells (ECs) (11). Currently, miR-424/503 has been identified as an important component of pulmonary vascular EC homeostasis, and miRNAs may serve as potential targets for treating PAH (12–15). HNRNPA2B1 may also contribute to the occurrence of PAH partly through these pathways. Although HNRNPA2B1 expression in pulmonary arterial endothelial cells (PAECs) is not significantly different from that in normal control cells (6), it may still exert its effects in PAH ECs by sorting and exporting specific miRNAs. In conclusion, HNRNPA2B1 shows great potential as a therapeutic target for PAH. We have comprehensively reviewed and presented the potential mechanisms through which HNRNPA2B1 drives the development of PAH.

## 2 HNRNPA2B1

HNRNPA2B1 is a member of the heterogeneous nuclear ribonucleoprotein (HNRNP) family. Structurally, HNRNPA2B1 consists of two RNA recognition motifs (RRMs) located at its N-terminus, referred to as RRM1 and RRM2, and a C-terminal low-complexity region rich in glycine residues, which includes an RGG box, a core PrLD domain, and an M9 nuclear localization signal (M9-NLS) (16). The RRMs and the RGG box are crucial for RNA binding (16, 17). HNRNPA2B1 can bind to specific m6A-modified regions on mRNA through its RRM domains, thereby mediating the translation and degradation of downstream mRNA (18). Although the RGG box significantly affects binding strength, its impact on specific RNA binding is relatively small (19). HNRNPA2B1 plays a role in mRNA splicing, mRNA modification, promotion of pre-mRNA synthesis, transport of mature mRNA, and maintenance of mRNA stability; therefore, its role spans almost the entire process, from mRNA synthesis to maturation (10, 20, 21). As an m6A reader, HNRNPA2B1 can recognize the m6A-specific motifs RGAC and interact with the microprocessor complex protein DGCR8, regulating selective splicing and processing of target mRNAs (10). HNRNPA2B1 may regulate the splicing and processing of target mRNAs by recognizing m6A, thereby activating or inhibiting downstream mechanisms involved in various pathophysiological processes.

HNRNPA2B1 was initially discovered as a tumor-associated antigen in non-small cell lung cancer (22, 23). Patients with non-small cell lung cancer who are positive for HNRNPA2B1 have a worse prognosis (24). HNRNPA2B1 is involved in various pathological and physiological processes, including inflammation (25), immunity (26), oxidative stress, cell proliferation, apoptosis (27), EMT (28), and metabolism (29). It is implicated in the development of various diseases such as cancers, rheumatic immune system diseases, neurological disorders, and viral infections (30). However, few studies have elucidated the relationship between PAH and HNRNPA2B1. Research suggests that HNRNPA2B1 may play a potential role in the underlying mechanisms of PAH. Pulmonary vascular remodeling is a basic pathological feature in all groups of PH. In PAH, the affected vessels are small arteries, whereas in conditions like pulmonary venous obstructive disease, pulmonary capillary hemangiomatosis, or PH-LHD, the medium-sized veins and capillaries are primarily involved. It is characterized by the accumulation of different vascular cells (PASMCs, PAECs, fibroblasts, myofibroblasts, and pericytes) in the pulmonary artery wall, as well as the disappearance of precapillary arterioles and excessive infiltration of inflammatory cells around the vessels, such as B and T lymphocytes, mast cells, dendritic cells, and macrophages (31). The underlying mechanisms include abnormal proliferation and migration of PASMCs, dysfunction of ECs, infiltration of inflammatory cells, activation of fibroblasts in the vascular adventitia, and accumulation of extracellular matrix, all contributing to structural changes in the vessel wall. As an RNA-binding protein, HNRNPA2B1 is involved in pathological processes such as cell proliferation, migration, angiogenesis, EMT, cell metabolism, and inflammation through mechanisms including the regulation of gene transcription, m6A modification, miRNA sorting, and exosome secretion. HNRNPA2B1 has been implicated in the pathological mechanisms of various cell types, including smooth muscle cells (6, 32, 33), endothelial cells (11, 34), fibroblasts (35), and macrophages (36). Therefore, HNRNPA2B1 may contribute to pulmonary vascular remodeling by modulating the pathological processes in these cells.

## 3 HNRNPA2B1 as a key factor in PAH

### 3.1 The role of HNRNPA2B1 in smooth muscle cells

#### 3.1.1 HNRNPA2B1 promotes PASMCs proliferation and phenotypic transformation

Microscopic remodeling of the pulmonary arteries is the hallmark pathological change in the progression of PAH. Excessive proliferation of PASMCs leads to thickening of the medial vascular layer. PASMC abnormalities are believed to be crucial in the early development of PAH. Normal PASMCs are highly differentiated cells with a contractile phenotype. However, in PAH, PASMCs undergo excessive proliferation, migration, and invasion, accompanied by increased extracellular matrix secretion, leading to a synthetic phenotype. This transition is known as phenotypic switching in PASMCs (38). In experimental PAH, including both MCT-induced and hypoxia-induced models, PASMCs undergo phenotype switching. Targeting phenotypic switching can improve preclinical pulmonary hypertension (39–41). Various signaling pathways, transcription factors, cytokines, and inflammatory mediators can trigger phenotypic switching of PASMCs. These details have been comprehensively discussed in previous reviews (42). The phenotypic switching of PASMCs ultimately results in pulmonary vascular thickening, loss of elasticity, distal vessel muscularization, and eventually vascular remodeling, particularly in the early stages of PAH (43). The dedifferentiation and proliferation of PASMCs can be regulated by signaling pathways such as STAT/ERK (7), PI3K/AKT/mTOR (44), AKT/STAT3 (27), ILF3/AKT (45), ERK/MAPK (46), Wnt-β/catenin (47), as well as epigenetic mechanisms like histone modification and DNA methylation, which control the phenotypic transformation of PASMCs (42). The treatment goal of PAH could also be to reverse or inhibit the abnormal proliferation and dedifferentiation of PASMCs.

**Figure 1:**
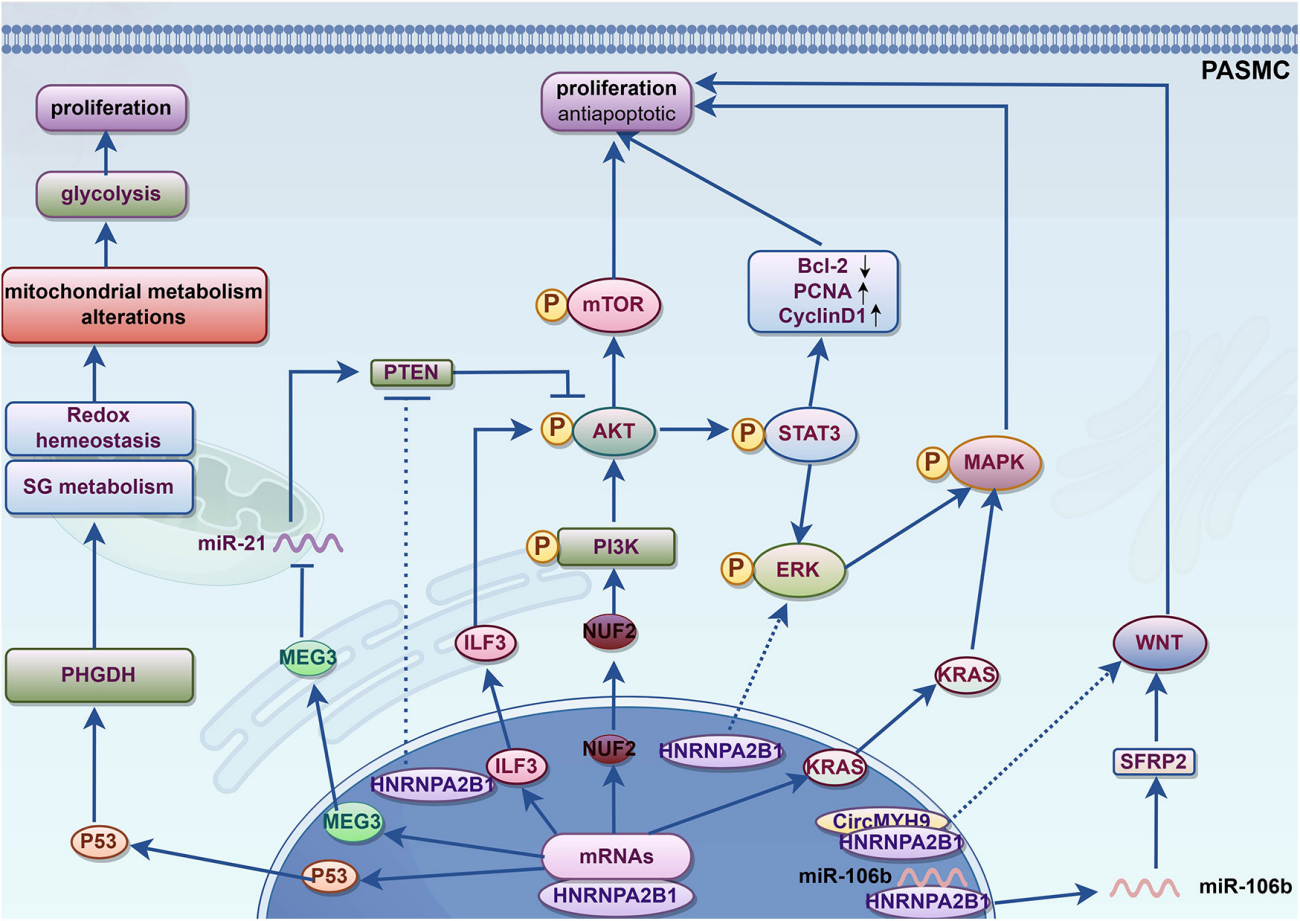
Potential mechanism of HNRNPA2B1 promotes phenotypic switching of PASMCs. KRAS, kirsten rat sarcoma viral oncogene homolog; MAPK, mitogen-activated protein kinase; ERK, extracellular regulated protein kinases; STAT3, signal transducer and activator of transcription; NUF2, Ndc80 kinetochore complex component; PI3K, Phosphoinositide 3-kinase; AKT, also known as protein kinase B or PKB; mTOR, Mammalian target of rapamycin; CyclinD1, G1/S specific cyclin D1; PCNA, Proliferating cell nuclear antigen; Bcl-2, B-cell lymphoma-2; ILF3, Interleukin enhancer binding factor 3; PTEN, Phosphatase and tensin homolog; MEG3, Maternally Expressed 3; PHGDH, Phosphoglycerate dehydrogenase; SG metabolism, serine/glycine metabolism; SFRP2, secreted frizzled related protein 2; WNT, Wingless/Integrated.

HNRNPA2B1 plays a role in vascular SMCs, including coronary artery, SMCs and PASMCs (48, 49). HNRNPA2B1 has the potential to regulate the phenotype of SMCs. HNRNPA2B1 can transcriptionally regulate the expression of SMCs genes by directly binding to the promoters of the Sm*α*a and Sm22α genes, its knockdown leads to the downregulation of specific smooth muscle markers and transcription factors, indicating its crucial role in SMC differentiation (50). Besides, HNRNPA2B1 can promote cell proliferation in various pathways, these mechanisms can also promote PASMCs proliferation (see Table 1). HNRNPA2B1 regulates the biological processes of multiple mRNAs and exerts biological effects, while being itself regulated by various factors. All these interactions results in a tight regulation of cellular metabolism. HNRNPA2B1 can modulate the cell cycle by regulating the expression of cell cycle-related proteins such as cyclin-dependent kinases and cyclin-dependent kinase inhibitors, thereby influencing cell metabolism (32). Additionally, HNRNPA2B1 can promote cell proliferation and invasion through mechanisms involving exosome sorting and regulating of various microRNAs (miRNAs) (47). Furthermore, it is involved in regulating transcription factors such as ZEB (36), as well as lipid synthesis (51), oxidative stress, and serine metabolism (52). In conclusion, HNRNPA2B1 may play a role in the phenotypic switching of PASMCs through multiple mechanisms. The mechanisms by which HNRNPA2B1 may affect the proliferation and phenotypic transformation of PASMCs were illustrated in Figure 1 and Table 1.

**Table 1:** Mechanisms by which HNRNPA2B1 may regulate PASMC metabolism.

Disease	Cells/Tissues	Upstream regulation	Role of gene overexpression	Role of gene knockout/knockdown	Mechanisms	Literatures	Literatures related to PH
PAH	PASMCs	-	-	↓Cell proliferation; ↑Apoptosis	↓Target mRNAs containing CUAGACUAGA, UAA[CG]UUAU, and GCC[GC]AAG[GA][AG][GA]CC motifs	(6)	-
Atherosclerosis	VSMCs	Down-regulated by lncRNA AC105942.1	-	Reversal VSMCs proliferation induced by Ang II	↓CDK4; ↑P27	(32)	(53)
Breast cancer	Breast cancer cell	-	-	↓Cell proliferation; ↑Apoptosis; ↑ S phase of the cell cycle	↓STAT3/ERK1/2	(7)	(54, 55)
Ovarian cancer	Ovarian cancer cell	-	-	↓NUF2; ↓Cell proliferation; ↑Apoptosis	↓PI3K/AKT/mTOR	(44)	(56)
Glioma	Glioma cell	-	-	↓Cell proliferation; ↑Apoptosis	↓AKT/STAT3	(27)	(55, 57)
Multiple myeloma	Multiple myeloma cells	-	↑Cell proliferation;↑ILF3/AKT	↓Cell proliferation; ↑Apoptosis; ↓ILF3/AKT	↑↓ILF3/AKT	(45)	(57, 58)
Colorectal cancer	Colorectal cancer cells	Interaction with circMYH9	-	↓Cell proliferation	↓P53; ↓Serine metabolism and ROS	(52)	(59)
Colorectal cancer	Colorectal cancer cells	-	-	↓Cell proliferation; ↑Apoptosis; ↑Cell cycle arrest	↓ERK/MAPK	(46)	(63)
Hepatocellular carcinoma	Hepatocellular carcinoma cells	Decreased by knockdown of PCAT6 mediated by miR-326	-	↓Cell proliferation and invasion	-	(60)	(64)
Adenocarcinoma of lung	Adenocarcinoma of lung cells	Up-regulated by INC00963	-	↓Cell proliferation, invasion and EMT	↓ZEB1	(36)	(65)
Nasopharynx cancer	Nasopharynx cancer cells	Decreased by knockdown of SOX2-OT mediated by miR-146b-5	↓Cell proliferation, ↑Cell proliferation	-	-	(61)	(66)
Colorectal cancer	Colorectal cancer cells	Stabilized by CRNDE mediated by inhibiting ubiquitination through TRIM21	↑Cell proliferation and migration; ↑KRAS/MAPK	↓Cell proliferation and migration; ↓KRAS/MAPK	↑↓KRAS/MAPK	(62)	(63)
Adenocarcinoma of the lung	Lung adenocarcinoma cells; embryonic kidney cells	-	-	↓Cell stemness, proliferation, migration and tumor growth	↓miR-106b-5p; ↑SFRP2; ↓Wnt-*β*/catenin	(47)	(72)
Non-small cell lung cancer	Non-small cell lung cancer cells	-	-	↓Cell proliferation and migration; ↓ MEG3 m6a; ↑MEG3 mRNA	↓miR-21-5p; ↑PTEN; ↓PI3K/AKT	(67)	(73)
Pancreatic cancer	Pancreatic cancer cells	Decreased by knockdown of Fyn	↓Bcl-x(s); ↓apoptosis	↑Bcl-x(s) and Bcl-x(s)/Bcl-x(L); ↑apoptosis	↓↑Bcl-x(s)	(68)	(74)
Esophagus cancer	Esophagus cancer cells	-	-	↓Adipogenesis; ↓Cell proliferation, migration and invasion	↓ACLY and ACC1	(51)	(75)
Esophagus cancer	Esophagus cancer cells	-	-	↓Cell proliferation	↓miR-17, miR-18a, miR-20a, miR-93 and miR-106b	(69)	(76–78)
Esophagus cancer	Esophagus cancer cells	Interaction with p53-G245S	-	↑Secretion of exosomes; ↑Cell proliferation	↓AGAP1	(70)	(59)
-	skeletal muscle cells	Up-regulated by miR-206 and lncRNA-lncA2B1	-	↓Cell proliferation	↓miR-206 and MyHC	(71)	(79)
Non-small cell lung cancer	Non-small cell lung cancer cells	-	-	↓Cell proliferation; ↑Apoptosis	↓ERK/p53/HDM2 and CKD2; ↑P21 and P27	(80)	(59, 63)
ovarian cancer	ovarian cancer cells	Decreased by knockdown of miR-30c-5p	-	↓Cell proliferation, migration and invasion	↓CDK19	(81)	(81)
Non-small cell lung cancer	Non-small cell lung cancer cells	-	-	↓Cell proliferation; ↑Apoptosis	c-Myc–LINC01234–HNRNPA2B1–miR-106b-5p–CRY2–c-Myc	(82)	(83)

#### 3.1.2 HNRNPA2B1 regulates cell metabolic reprogramming

Metabolic reprogramming refers to the alteration of cellular metabolism in response to various stress conditions. Under normal circumstances, the main energy source for cells is oxidative phosphorylation through the tricarboxylic acid cycle (TCA cycle). However, in PAH, PASMCs shift their energy production toward glycolysis, a phenomenon known as the “Warburg effect”. During the Warburg effect, cells exhibit increased cytoplasmic glycolysis and glutaminolysis, while mitochondrial biogenesis and fatty acid oxidation are inhibited. Consequently, PASMCs in PAH primarily rely on glycolysis for energy production to support their growth. The promotion of glycolytic metabolism through metabolic reprogramming enhances PASMC survival and proliferation.

Abnormal PASMC metabolism may serve as a potential therapeutic target for PAH, with preliminary success observed in clinical trials. TEPP-46, an activator of pyruvate kinase M2 (PKM2), has been shown to normalize glycolysis and mitochondrial abnormalities in fibroblasts from PAH patients (84). Notably, PKM2 inhibitors reduce pulmonary artery pressure in Group 2 PH and reverse pulmonary vascular remodeling (85). Furthermore, PKM2 is highly expressed in PASMCs in Group 1 and Group 4 PH and accelerates their abnormal proliferation (86, 87). Interestingly, as a member of the HNRNP family, HNRNPA1 inhibition can downregulate PKM2 expression (87). Additionally, HNRNPA2B1 regulates PKM2 splicing, upregulates PKM2 expression, and subsequently modulates cellular metabolic reprogramming (29, 88). In an acute myocardial infarction model, shikonin inhibited HNRNPA2B1 activity and decreased PKM2 expression, thereby improving post-infarction inflammation, apoptosis, and fibrosis (89). These studies suggest that HNRNPA2B1, as an RBP, may regulate PASMC metabolic reprogramming by modulating PKM2 mRNA expression.

### 3.2 HNRNPA2B1 regulates EC function through exosome sorting

HNRNPA2B1 promotes cell proliferation and inhibits apoptosis. While tumor cells have been the main focus of studies on HNRNPA2B1, fewer studies have focused on its effects on SMCs, and even less evidence exists regarding its role in ECs. No significant difference in HNRNPA2B1 expression has been observed in PAECs from PAH mouse models (6), however, it is speculated to regulate miRNA sorting into exosomes, thereby modulating EC function through intercellular signaling. Notably, exosome therapy has shown promising results in experimental PAH (90). SMCs can promote the release of specific miRNA-loaded exosomes under hypoxia or TGFβ1 stimulation, which regulate EC metabolism through intercellular crosstalk (83, 91). Macrophage immune regulation plays an important role in PAH, and exosome therapy using mesenchymal stem cells has been shown to be effective in PAH animal models (90). miR-503 plays a critical role in PAEC proliferation and metabolism, not only inhibiting their proliferation but also exerting paracrine effects on PASMCs to suppress their proliferation and migration (12). In human umbilical vein endothelial cells (HUVECs), HNRNPA2B1 negatively regulates miR-503 exosomal sorting by inhibiting its secretion. However, in the presence of beraprost sodium, HNRNPA2B1 translocates to the nucleus and promotes miR-503 exosomal secretion, thereby exerting anti-tumor effects (11). In PAH, miR-503 overexpression inhibits ERK1/2 phosphorylation via targeting FGF2 and FGFR1, subsequently suppressing cell proliferation (12). Platelet-derived growth factor (PDGF) induces miR-185 expression in SMCs, and HNRNPA2B1 binds to the “GGAG” exosomal motif within miR-185. Through exosome transfer to ECs, this complex targets CXC motif chemokine ligand 12 (CXCL12), inducing angiogenesis. Importantly, HNRNPA2B1 inhibition significantly reduces neovascularization (49). These findings suggest that exosomes carrying functional miRNAs mediate crosstalk between ECs and SMCs.

HNRNPA2B1 can also promote the exosomal sorting of miR-122-5p (92, 93), miR-934, and lncRNA H19 (94). miR-122-5p is upregulated in hypoxia-induced pulmonary microvascular endothelial cells (PMECs), as well as in the lung tissues of SuHx rats, MCT rats, and IPAH patients. It may contribute to the pathogenesis of IPAH through the regulation of dihydrolipoamide S-acetyltransferase (DLAT) and regulating synaptic membrane exocytosis 1 (RIMS1), although the exact mechanisms require further investigation (95). Additionally, miR-122-5p regulates fatty acid utilization through 1-acylglycerol-3-phosphate O-acyltransferase 1 (AGPAT1) and promotes vascular development in ECs (96). miR-934 is transferred from tumor cells to macrophages via exosomal sorting, where it promotes macrophage polarization through the PTEN/PI3K/AKT signaling pathway. LncRNA H19 promotes the proliferation and migration of colorectal cancer cells through the Raf/ERK signaling pathway (97). Notably, H19 expression is significantly elevated in the pulmonary artery endothelium, and its deficiency improves pulmonary vascular remodeling. These effects are linked to the inhibition of EndMT via the TGF-β signaling pathway (98). Previous studies have established the role of these signaling pathways in EndMT.

**Figure 2:**
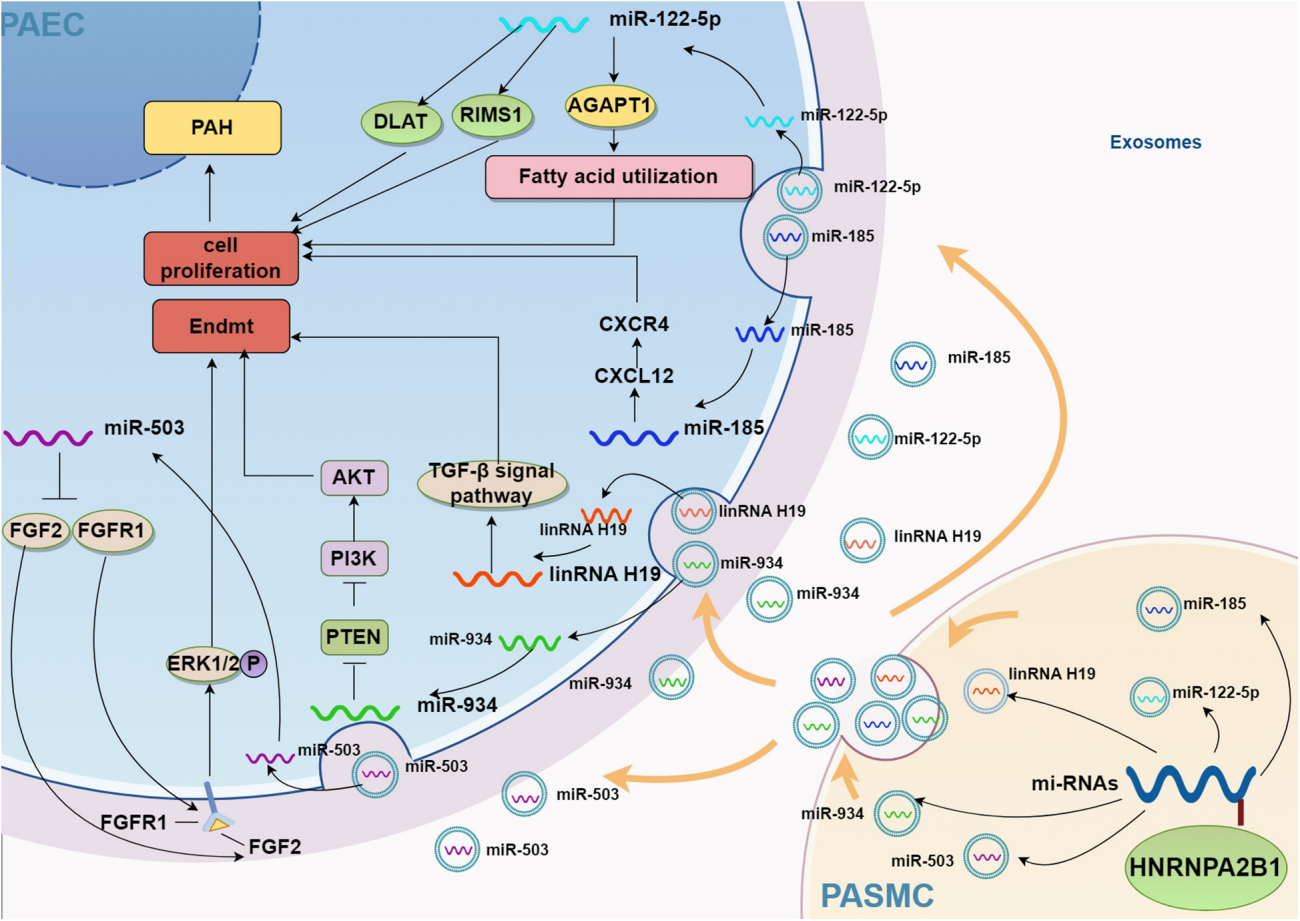
Potential mechanism of HNRNPA2B1 regulating exosome secretion. AGPAT1, 1-acylglycerol-3-phosphate O-acyltransferase 1; CXCL12, CXC motif ligand 12; CXCR4, CXC motif chemokine receptor 4; PTEN, phosphatase and tensin homolog; AKT, also known as protein kinase B or PKB; PI3K, phosphoinositide 3-kinase; ERK1/2, Extracellular regulated protein kinases 1/2; DLAT, dihydrolipoamide S-Acetyltransferase; RIMS1, regulating synaptic membrane exocytosis 1; PAH, pulmonary artery hypertension; Endmt, endothelial to mesenchymal transition; FGF2, fibroblast growth factor 2; FGFR1, fibroblast growth factor receptor 1; PAEC, pulmonary artery endothelial cell; PASMC, pulmonary arterial smooth muscle cell.

In summary, exosomes released by SMCs, mesenchymal stem cells, and other cell types under pathological conditions can act on ECs through intercellular communication, with HNRNPA2B1 functioning as a key regulator of miRNA-loaded exosome sorting. Therefore, HNRNPA2B1 may regulate EC function by modulating the secretion of specific miRNAs via exosomal sorting mechanisms. The mechanisms through which HNRNPA2B1 regulates endothelial cell function via exosome-mediated signaling were illustrated in Figure 2.

Inflammation plays a crucial role in the onset and progression of cardiovascular diseases (99, 100), including PAH (101). In lung biopsies of PAH patients, multiple inflammatory cell types—macrophages, mast cells, T lymphocytes, B lymphocytes, dendritic cells, and neutrophils—have been detected around the remodeled pulmonary vascular system (37, 102). Macrophages are pivotal in the inflammatory processes underlying pulmonary hypertension. Consequently, targeting inflammation and immunity has become a major therapeutic strategy for PAH management. However, the exact role and mechanism of anti-inflammatory therapy in PAH remain unclear. Reports indicate that during inflammation and hypoxia, circulating monocytes are recruited to the lungs, replace resident stromal macrophages, and contribute to pulmonary vascular remodeling (103). These recruited cells differentiate in response to microenvironmental changes during lung injury or hypoxic exposure. Early in hypoxia, macrophages accumulate around pulmonary vasculature, exhibit a hypoxic response, and release pro-inflammatory cytokines. Subsequently, perivascular macrophage accumulation decreases, and the cells adopt a tissue-repair and anti-inflammatory phenotype (104). Polarized macrophages drive PAEC dysfunction, PASMC proliferation, and activation of pro-inflammatory fibroblast phenotypes by coordinating pro- and anti-inflammatory mediators (105). Studies demonstrate that HNRNPA2B1 promotes macrophage polarization through multiple mechanisms. Under IL-4/IL-13 stimulation, lincRNA-MIR99AHG is upregulated in macrophages, translocates to the nucleus, and binds to HNRNPA2B1, thereby promoting polarization (106). In models of intestinal inflammation and obesity, HNRNPA2B1 exacerbates inflammation by enhancing mRNA stability of pro-inflammatory genes (e.g., TNF-α, IL-6, IL-1β), positioning it as a therapeutic target (25). Beyond direct macrophage regulation, HNRNPA2B1 modulates macrophage function by enhancing exosome secretion from other cell types. For example, in breast cancer, HNRNPA2B1 mediates exosomal sorting of miR-184-3p; upon macrophage uptake, this miRNA targets EGR1 to inhibit JNK signaling and induce polarization (107). In glioma, HNRNPA2B1 packages circNEIL3 into exosomes, which are delivered to tumor-associated macrophages to promote progression (108). Furthermore, neddylation-mediated degradation of HNRNPA2B1 downregulates mitochondrial trifunctional enzyme subunit α (MTPα), inhibiting NF-κB activation and inflammatory pathways (109).

**Figure 3:**
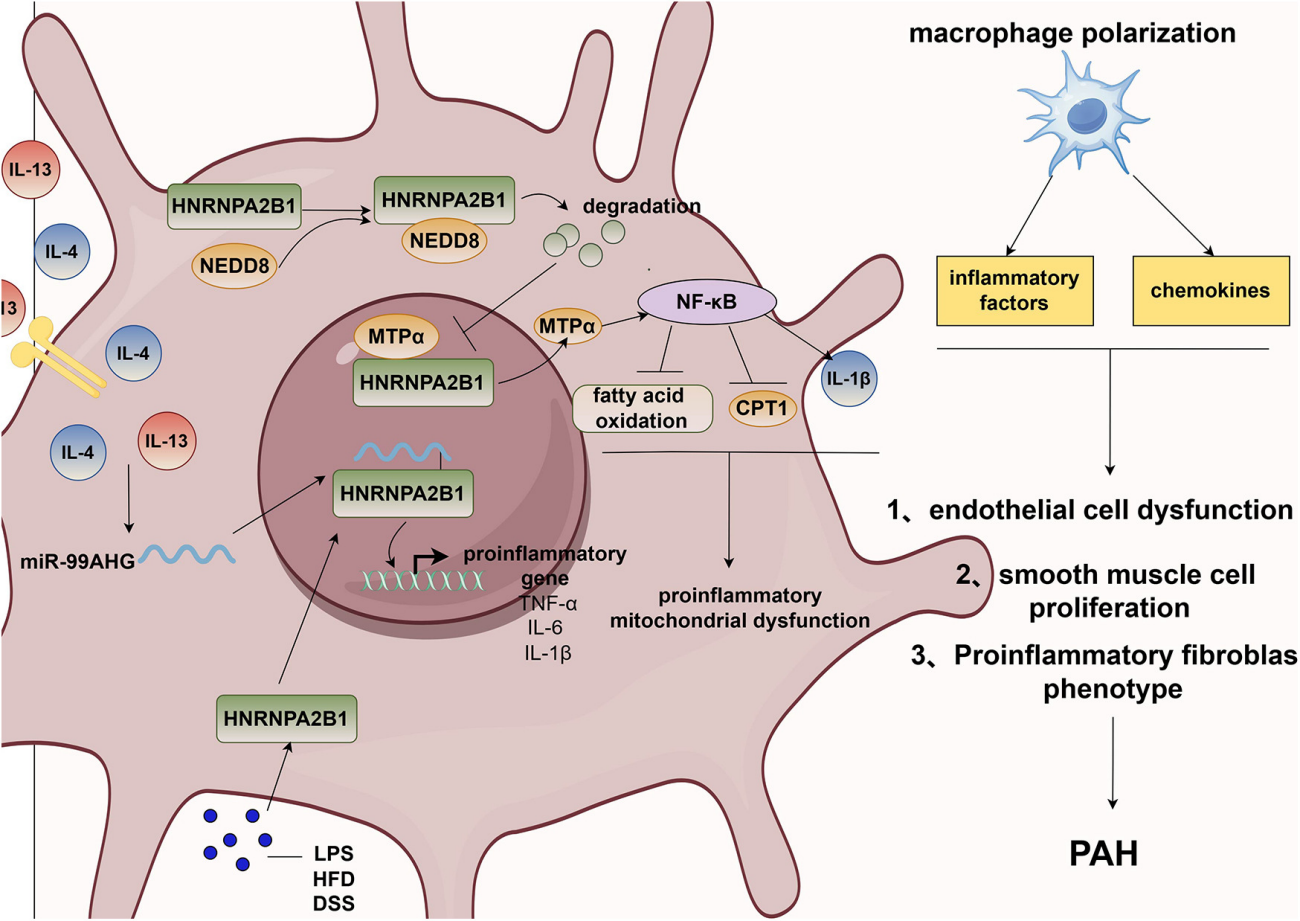
HNRNPA2B1 promotes pulmonary hypertension via macrophage polarization. PAH, pulmonary artery hypertension; IL-13, interleukin 13; IL-4, interleukin 4; NEDD8, neural precursor cell expressed developmentally down- regulated 8; MTPα, mitochondrial trifunctional protein α; CPT1, carnitine palmitoyltransferase 1A; NF-κB, nuclear factor kappa B subunit; TNF-α, tumor necrosis factor-α; IL-6, interleukin 6; IL-1β, interleukin 1β; LPS, lipopolysaccharides; HFD, high-fat-diet; DSS, dextran sodium sulfate.

In summary, the role of HNRNPA2B1 in inflammation remains unclear; however, as an RNA-binding protein (RBP), it regulates the expression of multiple RNAs and participates in exosome-mediated sorting processes. Given that inflammation is a complex process, a delicate balance between pro- and anti-inflammatory mechanisms exists under normal physiological conditions. However, when this balance is disrupted, pathological changes in the pulmonary vasculature occur. Therefore, the mechanisms of HNRNPA2B1 in inflammation may involve its regulation of both pro- and anti-inflammatory signaling pathways. In conclusion, HNRNPA2B1 may regulate the initiation and progression of pulmonary hypertension through inflammatory signaling pathways. Figure 3 illustrates how HNRNPA2B1 contributes to pulmonary hypertension via macrophage-mediated regulation of inflammation.

### 3.3 Potential role of HNRNPA2B1 in orther types of cells

In addition to the aforementioned cell types, fibroblasts and platelets also play roles in the onset and progression of PAH. Upon activation, fibroblasts exhibit excessive proliferation and reduced apoptosis, accompanied by upregulated inflammatory cascades and metabolic reprogramming. These processes may drive the migration of myofibroblasts to the tunica media or intima, contributing to vascular wall thickening (110). Fibroblasts further disrupt the balance between extracellular matrix (ECM) protein production and degradation, thereby promoting ECM remodeling. Activated platelets, on the other hand, form white thrombi at sites of vascular intimal injury, exacerbating thrombosis and PAH progression (111). Additionally, they secrete vasoconstrictors, growth factors, and inflammatory mediators to further promote PAH development (112). However, the relationship between HNRNPA2B1 and fibroblasts or platelets remains poorly understood. Bioinformatics analyses have shown no significant difference in HNRNPA2B1 expression between PAH tissues and controls (113). Thus, further studies are required to investigate whether HNRNPA2B1 contributes to PAH pathogenesis by activating fibroblasts and platelets.

### 3.4 Therapeutic prospects of targeting HNRNPA2B1 in PAH

HNRNPA2B1 can serve as a potential therapeutic target for various diseases due to its biological functions. Several compounds that target HNRNPA2B1 exert therapeutic effects through pathways implicated in the development of PH. Table 2 shows the therapeutic prospect of Targeting HNRNPA2B1 in diseases. The broad-spectrum antiviral drug PAC5, for example, binds to a pocket near the Asp49 RNA recognition motif (RRM1) of HNRNPA2B1, inducing its translocation into the cytoplasm and activating the TBK1-IRF3 pathway to exert antiviral effects (114). In the tumor microenvironment, the natural compound Sanggenol-O and its synthetic derivative MO-460 inhibit hypoxia-inducible factor 1 alpha (HIF-1α) expression via HNRNPA2B1, thereby triggering apoptosis (115), highlighting their potential as anticancer agents.

**Table 2:** Therapeutic prospects of targeting HNRNPA2B1 in diseases.

Medicine	siRNA	Target	Mechanisms	Diseases	Literatures	Literature related to PAH
PAC5		HNRNPA2B1	Transfer it into the cytoplasm, ↑TBK1-IRF3	HBV, COVID-19	(114)	-
Sanggenol-O,MO-460	-	HNRNPA2B1	↓HIF-1*α*, ↑apoptosis.	Cancer	(115)	(122)
Tripterygium wilfordii	-	HNRNPA2B1	↓PI3K-AKT, ↓cell proliferation	Cancer	(118)	(119)
Apigenin	-	HNRNPA2B1	↓proliferation of PASMCs, ↑apoptosis	PAH	(121)	(120)
-	HNRNPA2B1	PTEN\AKT\mTOR	↓proliferation, ↑apoptosis	CAD	(48)	-
-	HNRNPA2B1	-	↓cell proliferation, ↑apoptosis	PAH	(6)	-

Animal experiments demonstrate that HNRNPA2B1 interference reverses pulmonary hypertension in MCT-induced rat models (6). For instance, overexpression of the anti-aging enzyme SIRT6 inhibits hypoxia-induced proliferation of human pulmonary artery smooth muscle cells (HPASMCs) by activating the HIF-1*α*/PDK4 signaling pathway (116). SIRT6 also forms complexes with HNRNPA2B1, DGCR8, and Drosha to regulate macrophage pyroptosis and suppress inflammation under high glucose conditions (117). Additionally, Tripterygium wilfordii has emerged as a potential therapeutic agent for PAH *(*118). Studies indicate that it inhibits tumor cell proliferation by destabilizing HNRNPA2B1 mRNA and suppressing the PI3K-AKT pathway (119). The natural flavonoid apigenin, which inhibits PASMC proliferation and induces apoptosis, shows promise as a preventive or therapeutic option for PAH (120). Apigenin binds to the C-terminal of HNRNPA2B1, preventing its dimerization and potentially modulating its role in apoptosis (121). These findings collectively suggest HNRNPA2B1 as a promising therapeutic target for PAH, though further experimental validation is required.

## 4 Summary and prospect

**Figure 4:**
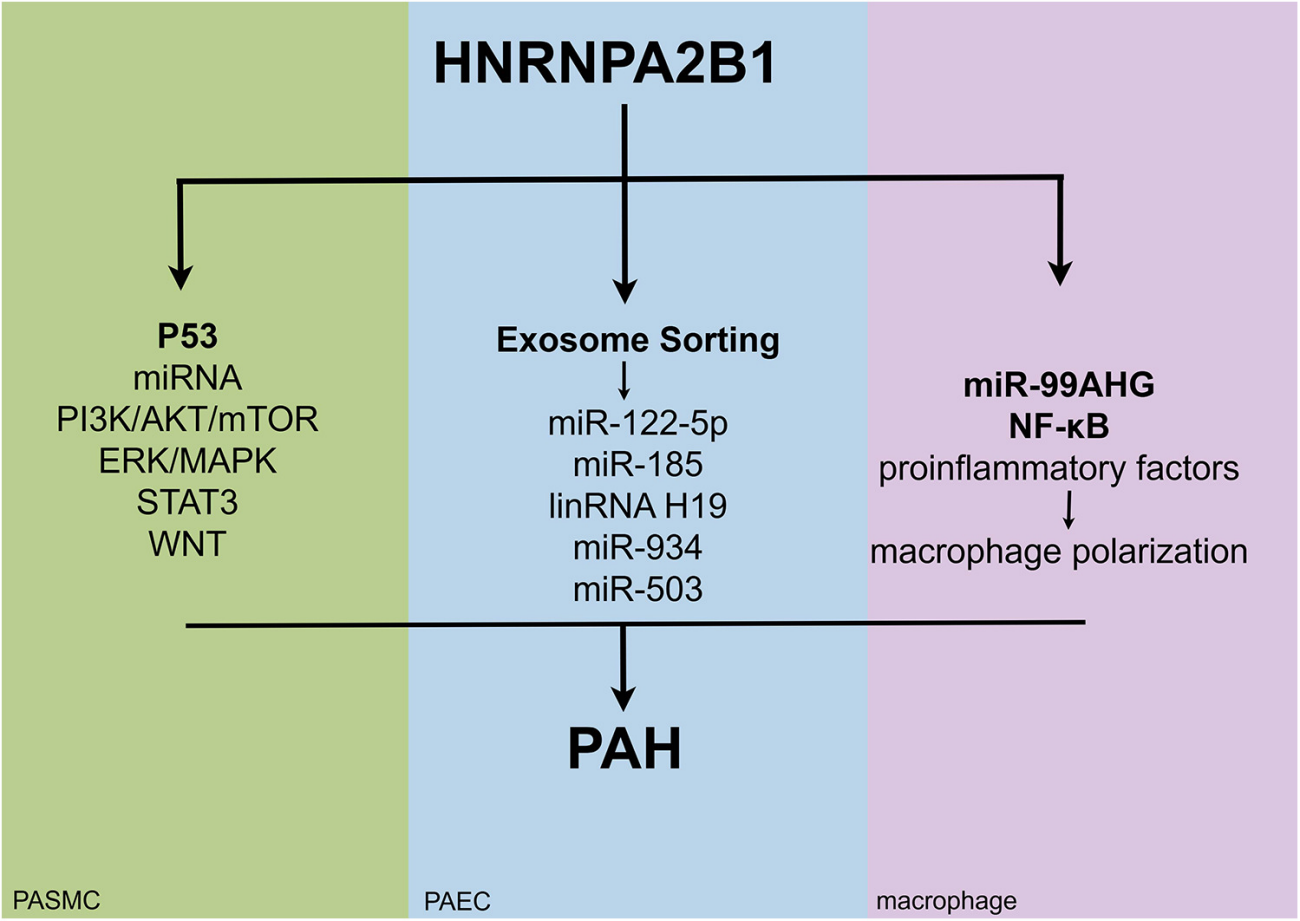
The potential mechanisms of action of HNRNPA2B1 in different cell types of pulmonary hypertension.

This review article highlights the potential mechanisms of HNRNPA2B1 in the pathogenesis of PAH. Figure 4 illustrates how HNRNPA2B1 may influence pulmonary hypertension by regulating PASMCs, PAECs, and macrophages. To date, only one study has demonstrated the role of HNRNPA2B1 in MCT-induced PH, though the specific mechanisms remain unexplored. Numerous studies suggest that HNRNPA2B1 is involved in cell proliferation, apoptosis, and PASMC metabolism (Figure 1); however, its exact mechanism of action in PASMCs is still unclear. The pathophysiology of PAH is complex, and the MCT-induced PH model fails to fully replicate the clinical features observed in PAH patients. Whether the proposed mechanisms can be generalized to other experimental models remains uncertain. Currently, no drugs targeting HNRNPA2B1 for PAH treatment exist. While gene knockdown models show promise in animals, their translational relevance to humans has yet to be established, underscoring the need for further research.

Additionally, the regulatory role of HNRNPA2B1 in EC function is poorly characterized. Although studies report no significant difference in HNRNPA2B1 expression in PAECs from PAH patients, it may still modulate exosome-mediated miRNA sorting mechanisms in ECs (Figure 2). Research on HNRNPA2B1-PAEC interactions in PAH remains in its early stages.

Structurally, the A2 and B1 isoforms of HNRNPA2B1 differ by only a 12-amino acid sequence, yet they exhibit distinct RNA-binding preferences. The ratio of these isoforms may vary across tissues and pathological stages, necessitating deeper functional characterization of this molecule (Figure 3).

Finally, HNRNPA2B1 regulates the Warburg effect and macrophage polarization—processes implicated in PAH progression. Nevertheless, the discussion of the relationship between HNRNPA2B1 and PAH is speculative, and there is currently no direct evidence to establish a connection between them. Therefore, further investigations are required to delineate its role in PAH mechanisms.

In summary, HNRNPA2B1 acts as an RBP through m6A-dependent mechanisms and regulates cell proliferation, apoptosis, metabolism, the immune microenvironment, and angiogenesis, all of which are important for the development of PAH. It is involved in the entire process, from mRNA generation to maturation, as well as in miRNA exosome sorting. However, the specific mechanisms need further confirmation. Targeted therapy involving HNRNPA2B1 is promising, but still in its early stages. Efforts to design and optimize treatment strategies targeting HNRNPA2B1 should continue.
